# Relation of Primary Fingerprint Patterns With Gender and Blood Group: A Dermatoglyphic Study From a Tertiary Care Institute in Eastern India

**DOI:** 10.7759/cureus.38459

**Published:** 2023-05-02

**Authors:** Ashok Rastogi, MD. Abu Bashar, Nishat Ahmed Sheikh

**Affiliations:** 1 Forensic Medicine, All India Institute of Medical Sciences, Patna, Patna, IND; 2 Community Medicine and Family Medicine, All India Institute of Medical Sciences, Gorakhpur, Gorakhpur, IND; 3 Forensic Medicine and Toxicology, All India Institute of Medical Sciences, Deoghar, Deoghar, IND

**Keywords:** fingerprints, relationship, forensic identification, gender, abo and rh blood groups, dermatoglyphics, biometrics

## Abstract

Background

Identification of an individual plays a vital part in any medicolegal investigation. The fingerprint is one of the oldest and most reliable biometric methods and is taken as legitimate proof of identification of an individual. Positive relationships between the fingerprint pattern and blood group and the fingerprint pattern and gender were demonstrated in some of the previous studies but weren't consistent across them.

Objectives

(a) To study the distribution of fingerprint patterns among the study participants by gender and ABO and Rh blood groups and (b) to find an association between the fingerprint pattern and gender and blood group.

Methods

A cross-sectional observational study was carried out in the year 2021 on 800 healthcare students and workers of All India Institute of Medical Sciences, Patna, Bihar, Eastern India having different ABO and Rh blood groups. Healthy individuals i.e., those who were not suffering from any illness which can affect the fingerprints, aged 18 years or above were included and individuals having hand or finger deformities or missing fingers, having an allergy to the ink pad, and having blood group diseases were excluded. Rolled imprints of all the 10 digits of the participants were taken on a white A4 size Performa and were classified into loops, whorl, arches, and composite. The distribution of the fingerprint patterns was then compared by gender, ABO and Rh blood group. Chi-square/Fischer exact tests were applied to compare two groups and find the association. P-value<0.05 was taken as statistically significant.

Results

The majority (66.0%) of the participants in the study were males with a male: female ratio of 1.9:1. Most common blood group was blood group B (37.7%) followed by O (29.8%), A (23.0%), and AB (9.5%). Rh-positive cases constituted around 96% of all the studied cases with the rest being Rh-ve. The general distribution of the fingerprint pattern showed a high frequency of loops registering 55.9%; followed by whorls (34.9%), arches (6.0%), and composite (3.1%). The distribution of fingerprint patterns among the male and female gender was found to be similar with no significant difference (p=0.11). However, the distribution of the finger patterns across the ABO blood groups showed a statistically significant difference (p=0.0003) whereas it was non-significant across the Rh blood groups (p=0.08).

Conclusion

This study concludes that the distribution of the primary fingerprint patterns relates to the “ABO” blood group but not to gender and Rh blood group. An individual's fingerprints may be used to predict his/her blood group and vice versa.

## Introduction

The use of characteristic features to ascertain the identity of a living or deceased individual is the hallmark of forensic science and the identification of an individual from his/her physical and mental attributes is a crucial objective of any forensic investigation. According to Locard's exchange principle, when two substances collide, some material is always exchanged from one to the other [1]. A person may carry traces from the incident away while also leaving traces at the place and the culprit of a crime will bring something into the crime scene and leave with something from it, and both can be used as forensic evidence [1].

Dermatoglyphics is the scientific study of naturally existing epidermal ridges and their organization on the digits, palms, and soles, in addition to the flexion crease and secondary folds. Anatomist Harold Cummins coined the term "dermatoglyphics" in 1926 after discovering that the shape of ridges on the sole and foot is influenced by genetics and an accidental or environmental effect during their creation in the intrauterine existence [2]. The pattern of dermal papillae decides the early development of the epidermal ridges. During the fetal period, the proliferation of the corium (dermis) forms papillary projections into the epidermis forming papillary ridges. The pattern of the papillary ridges in the hands is completely established between the 11th and 24th weeks of gestation [3]. These features, once completely formed, remain permanent and consistent throughout the life of an individual in all aspects except in their dimensions, to commensurate the growth of an individual postnatally [4]. Person identification using fingerprint algorithms is a well-established technique and is being used all over the world for security and authentication purposes. Over the past 150 years, dermatoglyphics has been proven as a useful tool in understanding basic questions in biology, medicine, genetics, and evolution, in addition to being the best and most widely used method for personal identification.

Even in monozygotic twins, dermatoglyphics is consistent and idiosyncratic from birth till death. A person's fingerprint is their unique identification [5,6]. Fingerprints are permanent individualized unique traits that are extremely detailed and difficult to change, acting as everlasting identifiers. A person's blood group, on the other hand, is a biological record that remains constant throughout one's life [7].

The following factors can be utilized for the identification of a person: (a) A person's possession (something he owns), such as a key to get physical access to a building, or information, such as "login access" to a system; (b) Positive identification is based on "BIOMETRICS", which includes physical characteristics such as fingerprints (dactylography), and behavioral/physiological characteristics such as voice, signature, and so on. In forensic sciences, biological characteristics like fingerprints and blood types are more dependable, authentic, and credible because they cannot be forged or duplicated [8].

Early detection of the perpetrator of the crime is a challenge in any criminal investigation with the available forensic tools. Many times, the only evidence left at the crime scene for the identification of the victim, or the crime perpetrator are fingerprints and some blood stains. The present study intends to identify the existence of a significant association, if any, between fingerprint patterns and gender as well as ABO and Rh blood groups. The study's findings would be of immense importance in the determination of gender and blood group from the fingerprints and vice versa, thereby improving the legitimacy of fingerprints in criminal investigations and the identification of criminals as well as victims with greater accuracy.

With this background, the present study was conducted among the healthcare workers of a tertiary care teaching institute in Eastern India to observe the predominant fingerprint pattern among them and to identify the relationship of fingerprint patterns with gender and ABO and Rh blood groups. The results of the study may also aid in creating local biometrics banks in the future as there is a paucity of research from this region of the country on finger patterns of the local population.

## Materials and methods

Study design and population

The current study was conducted among 800 faculty members, medical and nursing students, and other healthcare staff who were either studying or working at the All India Institute of Medical Sciences (AIIMS), Patna, Bihar, Eastern India.

Inclusion Criteria

Healthy individuals i.e., those who were not suffering from any illness which can affect the fingerprints, aged 18 years or above were included.

Exclusion Criteria

Individuals having hand or finger deformities or missing fingers, having an allergy to the ink pad, and having blood group diseases were excluded.

All the eligible participants were then invited to participate in the study and only those who gave written informed consent were included in the study. A universal sampling technique was used to enroll the study participants.

Study procedure

A proforma was printed on thick white paper, rubber stamp ink pads were used for smearing each finger, imprints were taken, and each fingerprint pattern was evaluated and documented using a powerful hand lens. The INK method, demonstrated by Cummins and Mildo in 1961 [9], was used to capture the fingerprints. A Faber-Castell blue color INK pad, A4-size white paper, cardboard, gauze pads, magnifying lens, pencil, and pen were used. Each proforma also included basic information about the participants such as name, gender, age, religion, and blood group, along with the fingerprints of all the 10 digits of the right and left hand. Each study participant was asked to wash his/her hands and dry them with the help of a clean towel. Subsequently, there were asked to press each of the right- and left-hand fingertips separately on the INK pad. Prints were taken on the white A4-size proforma. Fingerprint patterns (loops, whorls, and arches) were examined using a magnifying lens. Individuals with known blood types were noted, and those who did not know their blood group were subjected to Karl Landsteiner's [10] conventional ABO blood grouping process. The fingerprints were immediately evaluated using Michael Kuchen's fingerprint classification system [11].

Statistical analysis

The data collected were entered in Excel spreadsheets and analyzed using Statistical Product and Service Solutions (SPSS) (IBM SPSS Statistics for Windows, Version 20, Armonk, NY). Continuous data were expressed as mean with standard deviations and discrete data were expressed as frequencies and percentages. Chi-square/Fisher exact tests were used to compare two or more proportions. The level of significance was set as p-value < 0.05.

Ethical considerations

This study was conducted per the principles of the Declaration of Helsinki and the approval of the Institutional Ethics Committee (IEC) of All India Institute of Medical Sciences (AIIMS), Patna was taken prior to the commencement of the study (approval letter no. 03/2019). Written informed consent was obtained from all the participants after explaining the study objectives.

## Results

Out of the 800 study participants, the majority (66.0%) were males with a male: female ratio being 1.9:1. The most common blood group among the participants was B+ve (35.8%) followed by O+ve (28.1%) with A-ve (0.5%) being the least common. There was no participant with the AB-ve blood group (0.0%). As per the Rh factor classification, around 95.9% of participants were Rh+ve with the rest being Rh-ve (Table 1).

**Table 1:** Distribution of study participants according to blood group and gender (N=800)

Blood groups		Male		Female		Total	
		N	%	N	%	N	%
A	A+	119	22.6	61	22.3	180	22.5
	A-	2	0.3	2	0.7	4	0.5
B	B+	183	34.7	103	37.7	286	35.8
	B-	7	1.3	8	2.9	15	1.9
O	O+	147	27.9	78	28.6	225	28.1
	O-	13	2.5	1	0.3	14	1.7
AB	AB+	56	10.6	20	7.3	76	9.5
	AB-	0	0.0	0	0.0	0	0.0
	Total	527	100.0	273	100.0	800	100.0

**Figure 1:**
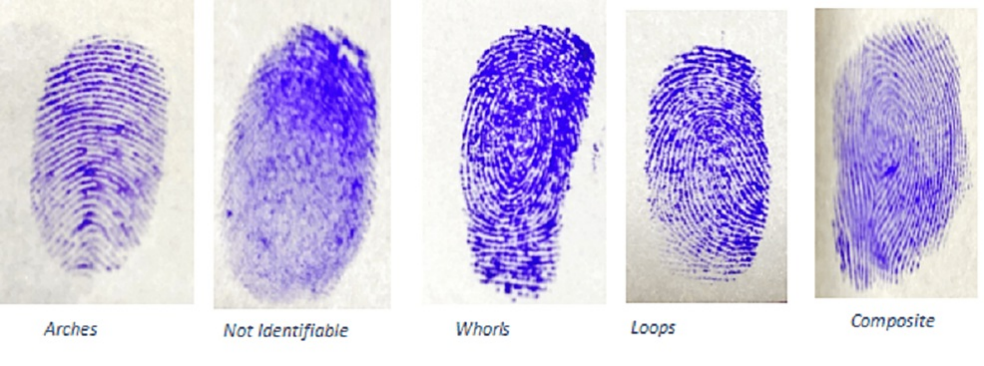
Examples of individual fingerprint patterns

The most common fingerprint pattern among the participants, overall, was loops (55.9%) followed by whorls (35.0%), arches (6.0%), and composite (3.1%). While comparing the fingerprint pattern between male and female participants, the loop pattern was the most common (55.7% v/s 56.4%) in both genders followed by whorls (36.0 v/s 32.9%). There was no significant difference found in the fingerprint pattern between males and females (p=0.11) (Table 2). Examples of individual fingerprint patterns from the study participants are shown in Figure 1.

**Table 2:** Distributions of primary fingerprint patterns in all fingers of both hands by gender (n=7133) *Chi-square test

Fingerprint patterns	Males		Females		Total		X^2*^-value, p-value
	N	%	N	%	N	%	5.94, 0.11
Loops	2935	55.7	1540	56.4	4475	55.9	
Whorls	1898	36.0	938	33.6	2796	34.9	
Arches	285	5.4	179	6.4	484	6.1	
Composite	152	2.9	73	2.6	245	3.1	
Total	5270	100.0	2730	100.0	8000	100.0	

With respect to the distribution of fingerprint pattern as per Rh blood group classification, loops were more common among Rh-ve individuals compared to the Rh+ve individuals (60.9% v/s 55.7%) whereas whorls were more common among Rh+ve individuals compared to the Rh-ve individuals (35.2% v/s 28.2%). However, the difference in the finger patterns between the Rh+ve and Rh-ve blood groups was statistically not significant (p=0.08) (Table 3).

**Table 3:** Distribution of fingerprints pattern in all fingers by Rh blood group (n=8000) *Chi-square test

Fingerprint patterns	Rh+ve blood groups n (%)	Rh-ve blood groups n (%)	X^2*^-value, p-value
Loops	4274 (55.7)	201 (60.9)	6.66, 0.08
Whorls	2703 (35.2)	93 (28.2)	
Arches	451 (5.9)	33 (10.0)	
Composite	242 (3.2)	3 (0.9)	
Total	7670 (100)	330 (100)	

With respect to the distribution of fingerprint patterns across the ABO blood groups, the highest proportion of loops was found in the AB blood group (61.0%) whereas it was least in the B blood group (52.4%). Similarly, whorls had the highest proportion in the B blood group (37.6% of the total) whereas it was the least in the AB blood group (30.8% of the total). The difference in fingerprint patterns across the ABO blood group was found to be statistically significant (p=0.0003) (Table 4).

**Table 4:** Distribution of primary fingerprints patterns in all fingers by ABO blood group (n=8000) * Statistically significant

Fingerprint patterns	Blood groups				
	A N (%)	B N (%)	O N (%)	AB N (%)	X^2^, p-value
Loops	1082 (58.8)	1577 (52.4)	1352 (56.6)	464 (61.0)	36.86, 0.0003*
Whorls	622 (33.8)	1132 (37.6)	808 (33.8)	234 (30.8)	
Arches	84 (4.7)	208 (6.9)	151 (6.3)	41 (5.4)	
Composite	52 (2.8)	93 (3.1)	79(3.3)	21 (2.8)	
Total	1840 (100)	3010 (100)	2390 (100)	760 (100.0)	

Table 5 depicts the distribution of the fingerprint patterns in the Rh+ve and Rh-ve individuals across each of the four ABO blood groups. The proportion of loops was higher among Rh-ve individuals across all the ABO blood groups whereas the proportion of whorls was higher among the Rh+ve individuals across all the ABO blood groups. No such pattern was observed in the case of arches and composite. Loops had the highest prevalence in the individuals with A-ve (67.5%) blood group followed by the O-ve blood group (61.8%). Whorls were most common among the B+ve blood group (37.7%) followed by the B-ve blood group (36.0%) whereas arches and composite were most common in O-ve (8.1%) and A-ve (5.1%) blood groups respectively. However, in none of the ABO blood groups, the difference in the fingerprint pattern between the Rh+ve and Rh-ve individuals was found to be significant (p>0.05).

**Table 5:** Distribution of primary fingerprint patterns in all fingers of both hands by ABO and Rh blood groups (n=8000)

Fingerprint patterns	Blood group A		Blood group B		Blood group O		Blood group AB	
	Rh + N (%)	Rh - N (%)	Rh + N (%)	Rh - N (%)	Rh + N (%)	Rh - N (%)	Rh + N (%)	Rh - N (%)
Loops	976 (58.6)	27 (67.5)	1318 (52.2)	57 (57.0)	427 (59.9)	84 (61.8)	464 (61.0)	0 (0.0)
Whorls	564 (33.9)	10 (25.0)	952 (37.7)	36 (36.0)	228 (32.0)	40 (29.4)	234 (30.8)	0 (0.0)
Arches	80 (4.8)	1 (2.5)	174 (6.9)	6 (6.0)	32 (4.5)	11 (8.1)	56 (7.4)	0 (0.0)
Composite	45 (2.7)	2 (5.0)	80 (3.2)	1 (1.0)	26 (3.6)	1 (0.7)	6 (0.8)	0 (0.0)
Total	1665 (100)	40 (100)	2524 (100)	100 (100)	713 (100)	136 (100)	760 (100)	0 (0.0)
Statistics	X^2^ = 2.68, p=0.45		X^2^ = 2.07, p=0.55		X^2 ^= 6.24, p=0.09		-	

## Discussion

Fingerprints are impressions made by the fine ridges, present at the fingertips, and are highly individualistic. The fingerprints are of key importance in establishing the identity of the culprits who were present at the scene of the crime along with the victims of mass disasters [12]. The system of classification for fingerprints, in use today, is adapted from the system proposed by Sir Francis Galton which was modified by Sir Edward Henry. The classification is known as the Henry Galton method or Henry’s system of classification [13]. This system of fingerprint classification is considered the most accurate and is in almost universal use.

The current study was carried out among 800 healthcare students and professionals of All India Institute of Medical Sciences (AIIMS), Patna; a tertiary care institute in Eastern India to study the pattern of fingerprints among them and to find out the relationship of fingerprints patterns with gender and blood group. To the best of our knowledge, this was the first study from this part of India that investigated the association of fingerprint patterns with gender and blood groups (ABO and Rh). The implications of finding a positive relationship between the identification techniques, fingerprints and gender, and blood group, will broaden the scope of biometric technology, which can offer identification solutions for personal and medical purposes, especially in the field of forensic medicine.

In our study, the majority of the participants were males (66.0%). The most common blood group was “B” followed by “O”, “A”, and “AB” blood groups, respectively, in both sexes. Similar results were reported by Patil et al. [14] from Navi Mumbai, Western India, Thakur et al. [15] from Bhopal, Central India, Mehta et al. [16] from Nagur, Central India, and Bhavana et al. [17] from Hubli-Dharwad of Karnataka, Southern India, where they observed the predominance of blood group “B” followed by blood groups “O” and “A" and the proportion of blood group “AB” was the least. The frequency of blood group “B” was also observed as the highest in the studies from Pakistan by Khalid and Qureshi [18] and from Iran by Ghasemi et al. [19]. On the other hand, studies by Bharadwaja et al. [20] from Ajmer, Northern India, Rastogi, and Pillai [21] from Mangalore, Southern India, Garg et al. [22] from Banda (U.P.), North India, Joshi et al. [23] from Solan, North India and Manikandan et al. [24] from Tamil Nadu, Southern India, Chaudhary et al. [25] and Kc et al. [26] from Nepal, and Fayrouz et al. [27] from Libya observed blood group “O” as most common followed by “B”, “A”, and “AB” blood groups.

In this study, the proportion of Rh+ve (95.9%) individuals was significantly higher across all the blood groups compared to Rh-ve (4.1%) ones. This is in congruence with the other studies done in various countries like India [14-17,20-23], Pakistan [18], Iran [19], Nepal [25,26], Libya [27], Nigeria [28], and Iraq [29].

The most common fingerprint pattern in our study, overall, was loops (55.9%) followed by whorls (35.0%), arches (6.0%), and composite (3.1%). Other previous studies from India [14-17,20-24] also found loops to be the commonest fingerprint patterns followed by whorls. Further, in our study, loops were the commonest fingerprint pattern observed both among males and females (55.7% and 56.4% respectively) followed by whorls (36.0% and 33.6% respectively) with no significant difference found between the two genders with respect to the fingerprint pattern. A study by Nithin et al. [30] from Southern India assessing fingerprint classification and its association with gender found ulnar loops to be the commonest fingerprint patterns followed by whorls both among males and females with no significant difference in finger pattern between the two genders, similar to our findings. Similarly, the study by Kc et al. [26] from Nepal also found loops followed by whorls to be the commonest fingerprint pattern both among males and females with no significant difference between the two genders. However, the study by Rastogi and Pillai [21] found that males have a higher incidence of whorls whereas females have a higher incidence of loops.

With respect to the association of the fingerprint pattern with the Rh blood groups, it was found that loops were commoner in Rh-ve (60.9%) individuals compared to Rh+ve (55.7%) ones whereas whorls were more frequently found among the Rh+ve individuals (35.2%) compared to the Rh-ve ones (28.2%). However, the difference was not found to be statistically significant. Similar results were reported in the studies by Patil et al. [14], Thakur et al. [15], and Kc et al. [26] where they found the incidence of loops to be higher among the Rh+ve individuals and the incidence of whorls to be higher among the Rh-ve individuals. However, in these studies too, this difference was not statistically significant implying fingerprint pattern is not related to the Rh blood group.

In our study, the prevalence of loops was highest in blood group AB (61.0%) whereas it was least in blood group B (52.4%). Similarly, the prevalence of whorls was highest among the blood group B (37.6%) whereas it was least in the blood group AB (30.8%). Studies by Patil et al. [14], Mehta et al. [16], Bhawana et. al. [17], and Kc et al. [26] also found loops to have the highest prevalence among the blood group AB and whorls to have the highest incidence among the blood group B.

With respect to the association of fingerprint patterns with the ABO blood group, in our study, the difference in the fingerprint pattern across the blood groups was found to be statistically significant implying an association of fingerprint patterns with ABO blood groups. Studies by Patil et al. [14], Bharadwaja et al. [20], Rastogi and Pillai [21], Garg, et al. [22], Joshi et al. [23], and Manikandan et al. [24] from India and Fayrouz [27] from Libya also found a significant association of fingerprint pattern with the ABO blood group. However, the studies by Chaudhary et al. [25] and Kc et al. [26] from Nepal found no significant statistical association between fingerprint patterns and the ABO blood group. This difference may be attributed to the difference in the study population of these studies.

In our study, Rh+ve individuals had a higher prevalence of loops whereas Rh+ve individuals had a higher prevalence of whorls across all four ABO blood groups. The prevalence of loops was highest among the individuals with the A-ve blood group (67.5%) followed by the O-ve blood group (61.8%) whereas the prevalence of whorls was highest among the individuals with the B+ve blood group (37.7%). However, in none of the ABO blood groups, there was a significant difference found in the fingerprint pattern between the Rh+ve and Rh-ve individuals. Similar findings were reported by Bhawana et al. [17] and Kc et al. [26] where the prevalence of loops was found to be higher in the Rh-ve individuals across all the ABO blood groups. However, in the study by Fayrouz et al. [27], the prevalence of loops was higher in Rh+ve blood groups O and A (54.3% and 52% respectively) than in whorls (33.4% and 30.6% respectively), somewhat not in line with our findings.

Limitations of the study

The somewhat small sample size and unequal sex distribution were the two main limitations of this study. Similar studies should be conducted on a larger sample with equal gender representation from this region to predict the more precise relationship of the dermatoglyphic patterns with gender and ABO and Rh blood groups.

## Conclusions

Loops are the most common fingerprint pattern, both among males and females and overall, followed by whorls with composite being the least common in the population of Eastern India. From this study's findings, it is concluded that the distribution of primary fingerprint pattern is related to the ABO blood group but not to gender and Rh blood group. The fingerprint pattern of an individual may be used to predict his/her ABO blood group and vice versa which can help in the identification of an alive/dead individual with greater accuracy. Since fingerprints will remain an essential part of the preferred biometric-based identification solutions in the coming years, a relationship of fingerprint pattern to the ABO blood group represents scope for additional identification data which can be utilized for personal identification purposes.
